# Best-evidence summary of pulmonary rehabilitation strategies for adult patients with cervical spinal cord injury: a systematic review

**DOI:** 10.3389/fmed.2026.1817133

**Published:** 2026-09-22

**Authors:** Ting Shao, Qiurong Lin, Xiaofei Chen, Wenjuan Zhou, Yaqin Dai

**Affiliations:** Center for Rehabilitation Medicine, Department of Orthopedics, Department of Nursing, Zhejiang Provincial People’s Hospital (Affiliated People’s Hospital), Hangzhou Medical College, Hangzhou, Zhejiang, China

**Keywords:** adult, cervical spinal cord injury, evidence-based nursing, health education, pulmonary rehabilitation, summary of evidence, systematic review

## Abstract

**Objective:**

To systematically sort out the relevant evidence on pulmonary rehabilitation in patients with cervical spinal cord injury (CSCI) at home and abroad, and provide evidence-based basis for clinical staff.

**Methods:**

The PIPOST (Population, Intervention, Professional, Outcome, Setting, Type of evidence) method was used to identify evidence-based questions. According to the “6S” pyramid evidence model, we systematically searched major Chinese and international databases, including MJ Best Practice, UpToDate, the Cochrane Library, PubMed, Web of Science, Embase, China National Knowledge Infrastructure (CNKI), Wanfang Data, and SinoMed. Inclusion criteria were adult patients (18 years or older) with CSCI and study types including expert consensus, clinical guidelines, evidence summaries, systematic reviews, randomized controlled trials (RCTs), and clinical decisions published in Chinese or English. Exclusion criteria were conference papers, guideline interpretations, and articles with unobtainable full text. Two team members independently completed quality assessment using corresponding evaluation tools from the Joanna Briggs Institute, extracted relevant evidence, and summarized and graded the evidence according to the Joanna Briggs Institute evidence grading system. The search time limit was from database establishment to December 31, 2025.

**Results:**

A total of 19 articles were included: 1 expert consensus, 1 guideline, 9 RCTs, 1 clinical decision, and 7 systematic reviews. After comprehensive analysis, 29 pieces of best evidence were extracted, covering 9 aspects including pulmonary rehabilitation team establishment, timing of rehabilitation, assessment methods, positioning management, and exercise training. Evidence level distribution: Level 1b (*n* = 2), Level 1c (*n* = 2), Level 2c (*n* = 4), Level 4 (*n* = 1), Level 5b (*n* = 14), and Level 5c (*n* = 1).

**Conclusion:**

The best evidence for pulmonary rehabilitation in patients with CSCI summarized in this study is scientific and practical, which can provide a reference for clinical practice.

**Systematic review registration:**

CRD420261376955.

## 1 Introduction

Cervical spinal cord injury (CSCI), also known as high-level spinal cord injury, is a complex neurological disorder that typically occurs in the upper segment of the spinal cord (1). It can lead to impairment of motor, sensory, and reflex functions below the level of injury, resulting in a range of clinical symptoms (2). According to statistics from the World Health Organization, CSCI accounts for approximately 51–55% of the annual incidence of spinal cord injuries globally, making it one of the most common types of spinal cord injuries. As a complex injury involving multiple systems, the impact of CSCI on the respiratory system is particularly significant. Following injury, the neural signal transmission of the phrenic nerve, intercostal nerve, and accessory respiratory muscles that control breathing may be disrupted, leading to respiratory dysfunction (3). The higher the level of injury, the more severe the involvement of respiratory muscles, and the greater the risk of respiratory complications in patients. Data indicate that the early mortality rate in CSCI patients due to respiratory complications is as high as 5.92%, with a disability rate of 50% (4, 5). Respiratory issues have thus become the leading cause of death in CSCI patients. Therefore, in clinical practice, the evaluation and intervention of respiratory function in adult CSCI patients are highly emphasized.

To effectively improve the respiratory function of CSCI patients and reduce the incidence of pulmonary complications, pulmonary rehabilitation has been proven to be an effective treatment method. Pulmonary rehabilitation is a comprehensive, interdisciplinary intervention program aimed at patients with respiratory symptoms and decreased daily living abilities. Its core objective is to alleviate symptoms, optimize respiratory function, and enhance overall quality of life through systematic rehabilitation measures (6). At present, there are some guidelines and expert consensuses on pulmonary rehabilitation after CSCI both domestically and internationally. However, these documents still lack clear recommendations in specific programs. For example, the optimal timing to initiate inspiratory muscle training, the criteria for progressing from supine to sitting position training, the standardized frequency and intensity for resistance breathing exercises during the acute phase, and the specific parameters for electrical stimulation therapy are not clearly specified. This lack of detailed guidance results in an insufficient reliable basis for clinical medical staff in actual operations. Of note, specific pulmonary rehabilitation protocols for adult patients with CSCI remain poorly defined in existing guidelines and expert consensus.

Therefore, this systematic review aims to retrieve and synthesize the best evidence on pulmonary rehabilitation exercise for adult patients with CSCI, so as to provide scientific and practical exercise guidance for clinical practitioners. This study was registered in PROSPERO (registration number: CRD420261376955).

## 2 Materials and methods

### 2.1 Problem establishment

Using the question-building tool developed by the Evidence-Based Nursing Center of Fudan University and based on the PIPOST (Population, Intervention, Professional, Outcome, Setting, Type of evidence) model, the initial question is constructed as follows: population (P): patients with CSCI; intervention (I): pulmonary rehabilitation-related interventions, such as respiratory muscle training and postural management; professional (P): clinical medical staff, rehabilitation therapists, nurses, etc.; outcome (O): respiratory function indicators, clinical symptoms, quality of life, etc.; setting (S): hospitals, rehabilitation centers, etc.; type of evidence (T): clinical decision-making, expert consensus, guidelines, randomized controlled trials (RCTs), systematic reviews.

### 2.2 Literature search strategy

Based on the “6S” evidence pyramid model (systems, summaries, syntheses, studies, synopses, and specials), a top-down search was conducted layer by layer. The search terms were “spinal cord injury/paraplegia/spinal fracture/spinal trauma/cervical spinal cord injury/quadriplegia” and “respiratory tract/respiratory management/pulmonary rehabilitation/respiratory function training/respiratory training/respiratory support.” The following databases were searched: MJ Best Practice, UpToDate, National Institute for Health and Care Excellence (NICE), Scottish Intercollegiate Guidelines Network (SIGN), Guidelines International Network (GIN), Registered Nurses Association of Ontario (RNAO), New Zealand Guidelines Group (NZGG), Medlive, Chinese Medical Knowledge Base, PubMed, Web of Science, Embase, Cochrane Library, Joanna Briggs Institute (JBI) OVID, China National Knowledge Infrastructure (CNKI), Wanfang Data Knowledge Service Platform, and Chinese Biomedical Literature Service System (SinoMed). The inclusion of Chinese databases (CNKI, Wanfang, and SinoMed) was specifically intended to capture studies on conventional pulmonary rehabilitation strategies as well as traditional Chinese medicine (TCM) pulmonary rehabilitation techniques. These TCM-based interventions are recognized as first-line adjunctive treatments for respiratory dysfunction in patients with CSCI within the Chinese healthcare system and are considered advantageous treatment options for this condition. However, most of these studies are published in Chinese journals and are not indexed in major international databases. Therefore, searching Chinese databases was essential to provide a comprehensive evidence summary reflecting real-world clinical practice in China. The search time was from the establishment of the database to December 31, 2025. Table 1 presents the search strategy for the PubMed database. Detailed search strategies for all other databases are provided in Supplementary Tables S1–S9.

**Table 1:** PubMed search strategy.

Serial number	Retrieval formula
#1	“Cervical Cord”[Mesh]
#2	“Spinal Cord Injuries”[Mesh]
#3	((((paraplegia[Title/Abstract]) OR (spinal fracture*[Title/Abstract])) OR (spinal trauma[Title/Abstract])) OR (cervical spinal cord injur*[Title/Abstract])) OR (tetraplegia[Title/Abstract])
#4	#1 OR #2 OR #3
#5	“Airway Management”[Mesh]
#6	“Respiration”[Mesh]
#7	(((((respiratory tract[Title/Abstract]) OR (respiratory management[Title/Abstract])) OR (pulmonary rehabilitation[Title/Abstract])) OR (respiratory function training[Title/Abstract])) OR (respiratory training[Title/Abstract])) OR (respiratory support[Title/Abstract])
#8	#5 OR #6 OR #7
#9	(((((((consensus[Title/Abstract]) OR (guideline[Title/Abstract])) OR (system evaluation[Title/Abstract])) OR (system review[Title/Abstract])) OR (evidence summary [Title/Abstract])) OR (clinical decision[Title/Abstract])) OR (best practice[Title/Abstract])) OR (meta-analysis[Title/Abstract])
#10	#4 AND #8 AND #9

### 2.3 Inclusion and exclusion criteria for literature

Following the PIPOST model to establish the research question, the inclusion and exclusion criteria for literature were formulated. Inclusion criteria: The subjects of the study were adult patients (≥18 years old) with CSCI; the types of studies included publicly published expert consensus, clinical guidelines, evidence summaries, systematic reviews, RCTs, and clinical decision-making, etc.; the language of publication was Chinese or English. Exclusion criteria: The types of literature were reports, guideline interpretations, and conference papers; literature with incomplete information or unobtainable full text; directly translated or repeatedly included literature; literature that did not pass the quality evaluation, including guidelines with less than 30% standardized percentage in two or more Appraisal of Guidelines for Research and Evaluation II (AGREE II) domains, RCTs rated as “high risk” of bias according to the Risk of Bias 2.0 (RoB 2.0) assessment, systematic reviews with “no” or “unclear” in four or more Joanna Briggs Institute (JBI) checklist items, and expert consensus with “no” in three or more items; non-Chinese or non-English literature; and outdated versions of guidelines that have been updated.

### 2.4 Quality assessment of literature

A literature quality assessment team was established, consisting of two main assessors who had undergone systematic training in evidence-based nursing, one rehabilitation specialist nurse from the Chinese Association of Rehabilitation Medicine, and one expert in evidence-based methodology. In cases where the evaluation opinions of the two main reviewers were inconsistent, all members of the group would participate in the discussion to reach a consensus conclusion. The tools for literature quality assessment are specifically described as follows: ① Guidelines: The quality of guidelines was assessed using the AGREE II. This system covers six major domains and 23 specific items, with each item rated on a scale from 1 to 7, where 1 indicates “strongly disagree” and 7 indicates “strongly agree.” The final quality score was comprehensively evaluated through the standardized percentage of scores in each domain. ② Expert consensus and systematic reviews: For expert consensus and systematic reviews, the methodological quality assessment tools developed by JBI were used for evaluation (7). The assessment tool for expert consensus contains six items, while the tool for systematic reviews contains 11 items. During the assessment, each item provides four response options: “yes,” “no,” “unclear,” and “not applicable.” ③ RCTs: The methodological quality of the included RCT was rigorously assessed by two independent reviewers using the Cochrane Collaboration’s recommended risk of bias assessment tool (RoB 2.0). The assessment systematically evaluated five key domains: (1) bias arising from the randomization process; (2) bias due to deviations from intended interventions; (3) bias due to missing outcome data; (4) bias in measurement of the outcome; and (5) bias in selection of the reported result. After assessment, each trial was classified as having an overall risk of bias of “low risk,” “some concerns,” or “high risk.” Any disagreements between the two reviewers were resolved through discussion to reach a consensus.

### 2.5 Evidence synthesis, extraction, and grading

During the process of literature screening and analysis, two independent researchers, respectively, summarized and organized the included literature. The specific operations included extracting the year, source, type, and research topic of the literature, and summarizing the relevant evidence according to the topic. When the evidence content was consistent or complementary, the researchers would summarize or integrate it; when the evidence content was relatively independent, its original expression was retained. If there were contradictions in the evidence from different sources, the researchers would prioritize evidence-based evidence, high-quality evidence, and the most recently published research results. Finally, based on the JBI evidence pre-grading system and its recommendation level system, the included evidence was graded (8).

Handling of missing information: When key data (e.g., sample sizes, effect sizes, standard deviations, specific *p-*values) were missing or incompletely reported in the included studies, we attempted to contact the corresponding authors via email to request the missing information. If no response was received within 4 weeks or the information remained unavailable, the data were marked as “not reported” and excluded from the corresponding quantitative synthesis (if applicable). For studies with missing full text, we made at least three attempts to obtain the article through institutional interlibrary loan services, direct contact with the authors, or other academic platforms (e.g., ResearchGate). If the full text remained unobtainable, the study was excluded from this review. All decisions regarding missing information were documented and reviewed by the two independent researchers, with disagreements resolved through discussion or consultation with a third reviewer.

## 3 Results

### 3.1 Literature screening results

A total of 443 relevant articles were identified through the literature search. After removing duplicates, 295 articles remained (148 duplicates excluded). After reading the titles and abstracts, 276 articles were excluded, with the main reasons being: (1) inconsistent research content (*n* = 67); (2) inconsistent study subjects (*n* = 61); (3) old literature (*n* = 53); (4) low quality of literature (*n* = 47); (5) unable to access full text (*n* = 32); (6) non-Chinese or English literature (*n* = 16). The remaining 19 articles were selected for full-text review. Following full-text evaluation, no additional articles were excluded, and 19 articles were finally included, comprising 1 expert consensus (9) 1 guideline (10), 9 RCTs (11–19), 1 clinical decision (6), and 7 systematic reviews (20–26). The specific process of literature screening is shown in Figure 1, and the basic characteristics of the included literature are summarized in Table 2.

**Figure 1:**
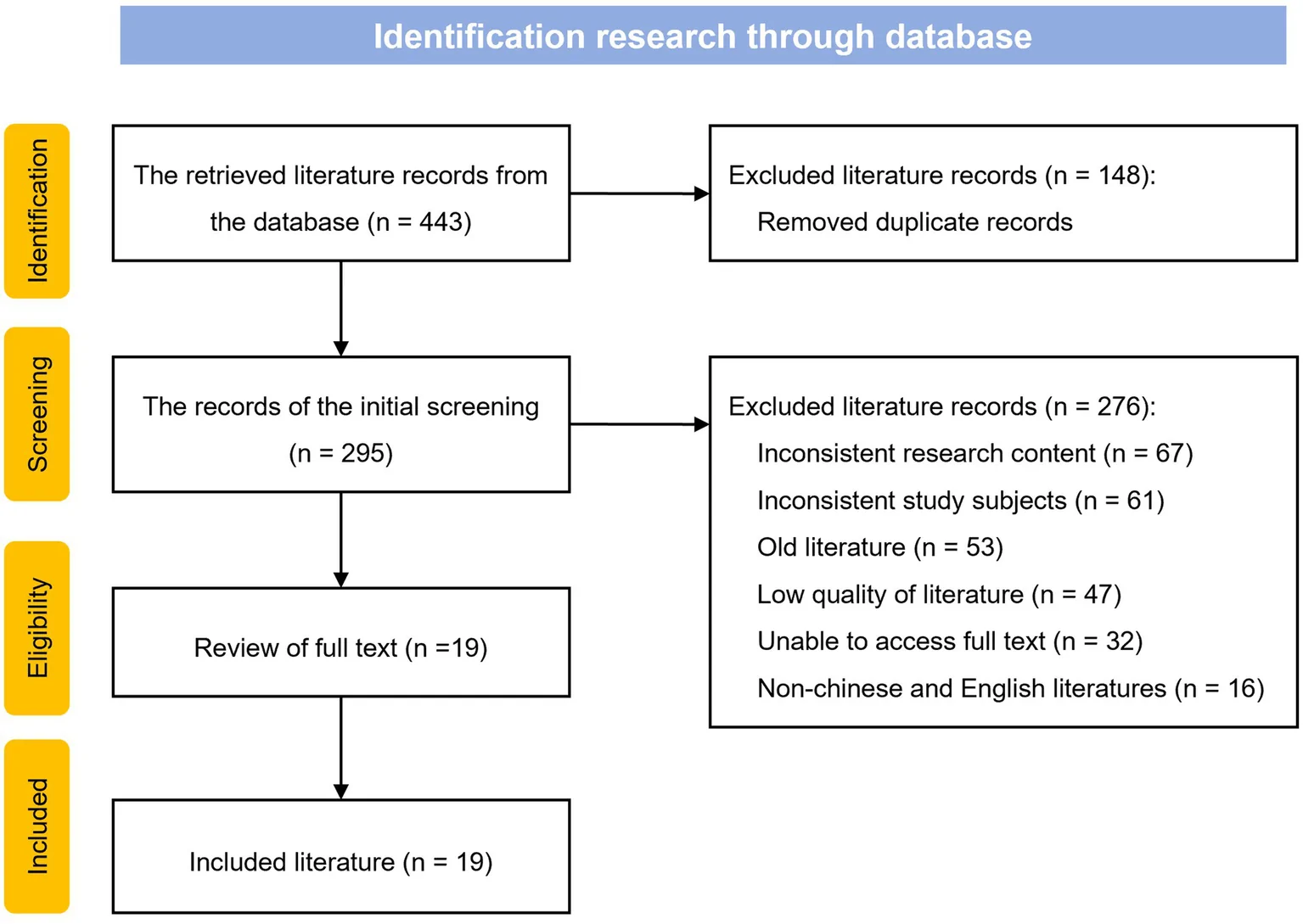
Literature screening process.

**Table 2:** Basic characteristics of the included literature.

Included literature	Year of publication	Topic of literature	Database	Type of literature
Liu et al. (9)	2022	Systematic management of traumatic spinal cord injury and common complications	CNKI	Expert consensus
Huo et al. (11)	2022	Effects of systematic respiratory training on diaphragmatic movement and pulmonary function in patients with CSCI	Weipu	RCT
Celli et al. (20)	2003	Respiratory system pulmonary rehabilitation	Up to date	Systematic review
Boswell-Ruys et al. (12)	2020	Effects of respiratory muscle training on respiratory muscles in patients with tetraplegia	Pubmed	RCT
Kim et al. (13)	2017	Effect of respiratory muscle training on pulmonary function decline in patients with CSCI	Up to date	RCT
Cui et al. (10)	2022	Rehabilitation treatment of spinal cord injury	Wan fang	Guideline
Fu et al. (14)	2014	Lung-function Training	CNKI	RCT
Park J et al. (6)	2022	Pulmonary rehabilitation training	Pubmed	Clinical decision
Hu et al. (21)	2023	Application progress of respiratory training in lung rehabilitation nursing of patients with CSCI	CNKI	Systematic review
Tsai et al. (15)	2024	Effects of craniocervical flexion and neck endurance exercises on pulmonary function in spinal cord injury	Pubmed	RCT
Liang et al. (16)	2021	The effect of narrative nursing combined with traditional Chinese medicine pulmonary rehabilitation training in improving the respiratory function of patients with spinal cord injury	CNKI	RCT
Xie et al. (22)	2017	Research Progress on the Application of Pulmonary Rehabilitation Concepts in the Nursing of Patients with CSCI	CNKI	Systematic review
Sharma et al. (17)	2021	The effect of glossopharyngeal blowing on the pulmonary function of patients with CSCI	Pubmed	RCT
Hill et al. (18)	2023	Pulmonary system functional and structural damage in middle-aged people with cervical and upper thoracic spinal cord injuries		RCT
Randelman et al. (23)	2021	Respiratory training and plasticity after CSCI	Pubmed	Systematic review
Rodrigues et al. (24)	2025	Respiratory muscle training for patients with CSCI	Pubmed	Systematic review
Chen et al. (25)	2025	A network meta-analysis of respiratory training methods for spinal cord injury patients	Pubmed	Systematic review
Hasnakipour et al. (19)	2025	Biofeedback diaphragmatic breathing for incomplete CSCI	Pubmed	RCT
Yao et al. (26)	2025	Respiratory muscle training for pulmonary function recovery in spinal cord injury	Pubmed	Systematic review

CNKI, China national knowledge infrastructure; CSCI, Cervical spinal cord injury; RCT, Randomized controlled trial.

### 3.2 Quality assessment results of the literature

#### 3.2.1 Quality assessment results of the guideline

One guideline, authored by Cui et al. (10) was included in this review. The recommendation grade was B. The detailed quality assessment results are presented in Table 3.

**Table 3:** Methodological quality assessment of the included guideline using the AGREE II instrument.

Guideline (year)	Scope and objectives	Persons involved	Rigor of development	Clarity of presentation	Applicability	Editorial independence	Standardized percentage (≥60%/≥30%)	Recommendation grade
Cui et al. (2022) (10)	68.05%	100%	78.12%	52.77%	20.83%	50%	3	2

AGREE II, Appraisal of guidelines for research and evaluation II.

#### 3.2.2 Quality assessment results of RCT

A total of 9 RCTs were included in this study. The methodological quality of each RCT was assessed using the Cochrane RoB 2.0 tool, which evaluates five domains and generates an overall risk of bias judgment (“low risk” or “some concerns”). The assessment results are summarized as follows: The studies by Kim et al. (13) and Boswell-Ruys et al. (12) were rated as low risk of bias across all five domains. The remaining seven studies Fu et al. (14), Huo et al. (11), Tsai et al. (15), Liang et al. (16), Sharma et al. (17), Hill et al. (18), and Hasnakipour et al. (19) were rated as some concerns in one or more domains, primarily due to issues related to randomization concealment, blinding of participants or outcome assessors, or incomplete outcome data. No trial was rated as high risk of bias. The detailed domain-level assessments for each study are presented in Figure 2.

**Figure 2:**
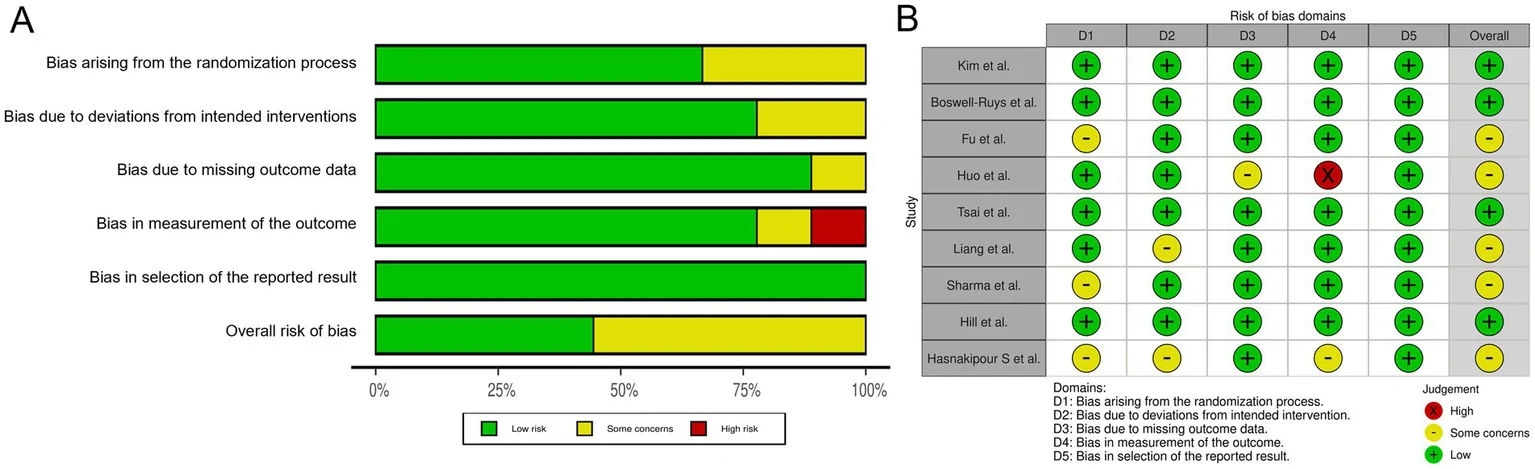
Risk of bias assessment of included RCTs. **(A)** Summary plot of risk of bias across studies for each domain. **(B)** Traffic light plot showing risk of bias assessment for individual studies.

#### 3.2.3 Quality assessment results of systematic reviews

A total of 7 systematic reviews were included in this study. In the study by Celli et al. (20), item 6 (“whether two or more reviewers independently assessed the quality of the literature”) was rated as “unclear,” while the remaining items were rated as “yes.” In the studies by Hu et al. (21), Xie et al. (22), and Randelman et al. (23), items 4 (“whether two or more reviewers independently screened the literature”), 5 (“whether two or more reviewers independently extracted data”), and 6 (“whether two or more reviewers independently assessed the quality of the literature”) were rated as “unclear,” while the remaining items were rated as “yes.” In the studies by Rodrigues et al. (24), Chen et al. (25), and Yao et al. (26), all items were rated as “yes.” All 7 systematic reviews were included in the final analysis (Figure 3).

**Figure 3:**
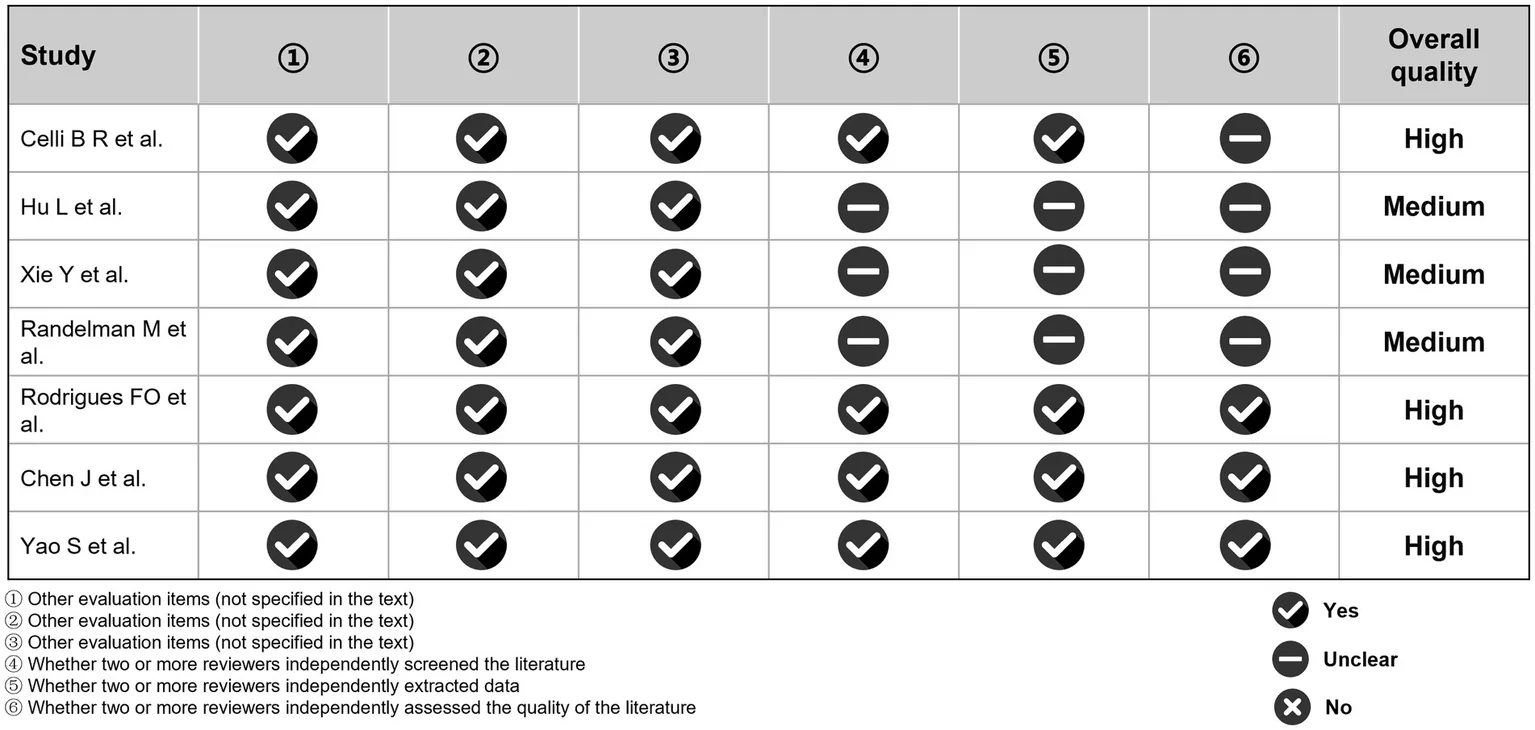
Quality assessment results of systematic reviews.

#### 3.2.4 Quality assessment results of expert consensus

One expert consensus was included in this study. In the study by Liu et al. (9), all items except item 4 “whether the conclusions are based on the analysis results and whether the expression of opinions is logical” were rated as “yes.” The overall quality was high and it was included.

#### 3.2.5 Evidence summary

Evidence was summarized for pulmonary rehabilitation in patients with CSCI, including nine aspects: establishment of a pulmonary rehabilitation team, timing of pulmonary rehabilitation, pulmonary rehabilitation assessment, position management, exercise training, improvement of pulmonary ventilation training, respiratory muscle training, training intensity and frequency, and health education. A total of 29 pieces of evidence were formed, which are shown in Table 4.

**Table 4:** Summary of the best evidence for pulmonary rehabilitation in adult patients with CSCI.

Evidence topic	Evidence content	Evidence level
Pulmonary rehabilitation team	1. Management by experienced rehabilitation physicians is required (9).	5b
	2. Participation of multidisciplinary medical and health professionals in rehabilitation treatment and care is required (20, 25).	5b
Content of pulmonary rehabilitation assessment	3. Assessment of patients’ vital signs, including Airway (A), Breathing (B), Circulation (C), and Spinal Cord (D) (14).	5b
	4. Assessment of spinal cord neurological function, including assessment of sensory function, motor function, and impairment grading (6, 14).	5b
	5. Early diagnosis and treatment of various complications (20).	5b
	6. Before participating in the pulmonary rehabilitation program, each patient should undergo an individual assessment to evaluate the severity of respiratory impairment, exercise tolerance, presence of comorbidities (especially cardiac, musculoskeletal, and neurological diseases), and cognitive-linguistic-psychosocial issues (20).	1b
	7. Assessment of medical history: level of spinal cord injury, severity of injury, presence of chest wall combined injuries, past history of pulmonary diseases, and smoking history (10).	5b
	8. Assessment of patients’ pulmonary function and diaphragmatic movement before training and 6 weeks after training (11).	1c
	9. It is recommended to use multiple indicators such as pulmonary function tests (e.g., FVC, FEV1, PEFR), diffusion function, and respiratory impedance to comprehensively assess pulmonary function (6, 18).	4
Pulmonary rehabilitation timing	10. Bedside training is the main focus during the acute phase (10).	5c
	11. Out-of-bed rehabilitation training is conducted during the recovery phase (14).	2c
Position management	12. For patients in the acute phase, adaptive training from the supine position to the sitting position should be conducted first (14).	5b
	13. Assist patients to assume a semi-recumbent position or wheelchair sitting position (14)	2c
Pulmonary rehabilitation training content	14. Singing training is used to train respiratory muscles and activate oral muscle movement at the same time; compared with conventional training, it can significantly improve pulmonary function (9).	5b
	15. Maintain the maximum inspiration posture for 3–4 s, then perform maximum expiration (13).	1c
	16. Oropharyngeal insufflation training can significantly increase pulmonary function indicators such as vital capacity, inspiratory capacity, and tidal volume (17).	2c
Respiratory muscle training	17. Conduct inspiratory muscle strength training for patients (9).	5b
	18. Respiratory training can start with slow and relaxed abdominal breathing, and gradually transition to resistance training for the diaphragm; at the same time, train the remaining sternocleidomastoid muscles and trapezius muscles to compensate for thoracic breathing (9, 11, 19).	5b
	19. Training includes abdominal breathing training, pursed-lip breathing training, low-frequency electrical stimulation of abdominal muscles combined with cough training, progressive inspiratory muscle resistance training, external diaphragm pacemaker therapy, active breathing cycle technique, and oropharyngeal breathing technique (23, 26).	5b
Training intensity and frequency	20. Twice a week of moderate-to-high intensity aerobic training, and at the same time, twice a week of 3 sets of strength training for major muscle groups such as the diaphragm, intercostal muscles, abdominal muscles, scalene muscles, and sternocleidomastoid muscles. The training plan needs to be tailored to individual conditions, with a training duration of 40 min per session, once a day, 5 times a week, for a total of 6 weeks (21, 24).	5b
Exercise training	21. As strength improves, the training intensity also increases, with 3–5 breaths per set, 12 times per set, and a 2-min interval of quiet breathing. Start with inspiratory training every day, and enter expiratory training after 2 min of calm breathing (20).	5b
	22. A total of 5 sets of respiratory training are conducted, with 10 repetitions per set; after each set, a 1-min rest is allowed (13).	1c
	23. Inspiratory training is generally 10–15 min per session, 3 times a day (14).	2c
	24. Resistance abdominal breathing training is 20 min per session, 3 times a day (14, 20).	2c
	25. Conduct music therapy (15, 22).	5b
	26. Respiratory training, sputum excretion training, postural drainage, and respiratory function training (10).	5b
	27. Increase chest wall movement; through deep breathing training, cough assistance, passive manual traction, joint movement methods, and intermittent positive pressure ventilation, etc., the range of chest wall movement can be maintained and improved (17, 22).	5b
Health education	28. Inform CSCI patients and their family members of the purpose and significance of respiratory training to improve patients’ compliance with respiratory training (10, 18).	5b
	29. Narrative nursing can alleviate anxiety and depression and improve patients’ active participation in respiratory rehabilitation (16).	2c

CSCI, Cervical spinal cord injury; FVC, Forced vital capacity; FEV1, Forced expiratory volume in one second; PEFR, Peak expiratory flow rate.

## 4 Discussion

CSCI has complex and systemic impacts on respiratory function. The underlying pathophysiological mechanism involves direct injury to spinal anterior horn motor neurons, respiratory-related neural pathways, and neuromuscular junctions. This triggers a series of pathophysiological changes, including weakened respiratory muscle contraction, disordered regulation of the respiratory center, and impaired airway defense function (27). Therefore, pulmonary rehabilitation for patients with CSCI should not be limited to conventional symptom-oriented management. Instead, it should adopt a comprehensive and multi-dimensional strategy that is based on pathophysiological mechanisms and targets multiple functional domains. The rehabilitation recommendations stratified by injury segment, severity, and evidence level are summarized in Figure 4.

**Figure 4:**
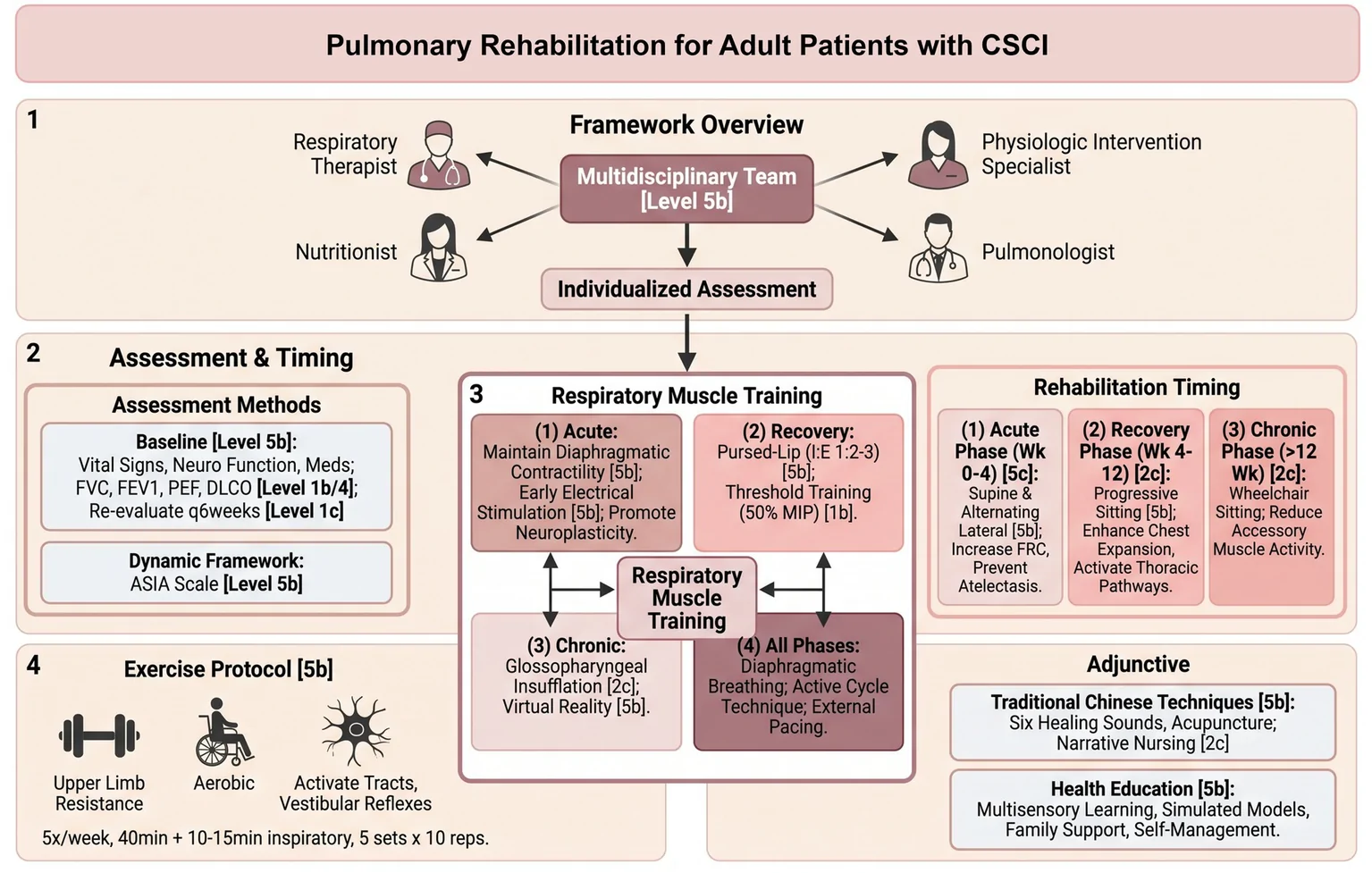
Summary of pulmonary rehabilitation strategies and evidence levels for adult patients with CSCI.

### 4.1 The importance of multidisciplinary teams in pulmonary rehabilitation for patients with CSCI

Evidence 1–2 included in this review was graded Level 5b, which represents low-quality evidence mainly obtained from expert consensus and observational studies. However, the implementation of a multidisciplinary rehabilitation team is strongly supported by the pathophysiological characteristics of CSCI. Respiratory dysfunction after CSCI involves disorders in neural regulation, respiratory muscle metabolism, and central integration, which cannot be effectively addressed by a single discipline (28).

From a clinical perspective, respiratory therapists optimize the compensatory mechanisms of residual respiratory neural pathways by adjusting ventilator parameters and using assisted expectoration devices, thereby improving the coordinated function of the diaphragm and accessory respiratory muscles. Occupational therapists provide scenario-based training grounded in neuroplasticity, which facilitates adaptive remodeling of the corticospinal pathways through repeated task-specific practice (21, 22). Orthopedic surgeons perform spinal stability assessments to provide a safe foundation for rehabilitation training; pulmonologists conduct dynamic monitoring to reflect improvements in ventilation–perfusion balance (15, 27); nutritional support sustains mitochondrial function and protein metabolism in respiratory muscles (16, 22); and psychological interventions regulate neuroendocrine pathways to reduce the inhibitory effects of stress on the respiratory center (15, 24).

Clinical data have confirmed that the multidisciplinary rehabilitation model is associated with a significant reduction in pulmonary complication rates, from 26.67 to 3.33%, validating the synergistic effects of multi-system interventions in breaking the pathological cascade of neural injury, muscle weakness, ventilatory impairment, and infection risk (29). Given its favorable safety profile and substantial clinical benefits, multidisciplinary pulmonary rehabilitation is therefore recommended as routine clinical practice for patients with CSCI. Since the current level of evidence remains low, future high-quality RCTs are needed to compare multidisciplinary rehabilitation with single-discipline approaches, so as to further strengthen and upgrade the evidence base.

### 4.2 Comprehensive and timely assessments are essential for pulmonary rehabilitation in CSCI patients

Evidence 3–9 in this study highlights that a systematic and dynamic assessment framework is essential to ensure the safety and effectiveness of pulmonary rehabilitation in patients with CSCI. Respiratory dysfunction after CSCI involves impaired neural conduction, metabolic disturbances of respiratory muscles, and disrupted neuromuscular regulation, requiring a multidimensional assessment approach.

Evidence 3–9 includes mixed levels of evidence. High-quality evidence (Level 1b) supports individualized baseline assessment using forced vital capacity (FVC), forced expiratory volume in 1 s (FEV1), peak expiratory flow rate (PEFR), diffusion capacity, and respiratory impedance, with reassessment at 6 weeks to guide training and evaluate outcomes. Lower-quality evidence (Level 4–5b) recommends supplementary assessments of vital signs, neurological function, medical history, and complications, which represent current best clinical practice despite being mainly based on expert consensus. Guided by neuroplasticity principles, the assessment framework integrates structural, functional, and adaptive dimensions (6). Baseline evaluation should clarify the relationship between injury level and respiratory muscle impairment. In patients with American Spinal Injury Association (ASIA) Impairment Scale (AIS) grade A complete injury, motor neuron loss leads to significant diaphragmatic dysfunction and restrictive ventilatory impairment (23). Dynamic monitoring including transcutaneous carbon dioxide and pressure–time index helps reflect ventilatory efficiency and respiratory muscle endurance, supporting individualized training intensity adjustment (23, 30). A tiered assessment strategy aligns with neural repair processes across acute, recovery, and chronic phases. Psychosocial and cognitive function should also be evaluated to improve rehabilitation adherence (18). Taken together, a pathophysiologically grounded multidimensional assessment system shifts pulmonary rehabilitation from symptom control to targeted neural functional reconstruction, which optimizes clinical outcomes and helps clarify the neurobiological mechanisms of respiratory recovery after CSCI.

### 4.3 Different stages of pulmonary rehabilitation require different timing

Evidence 10–11 in this review was graded Level 2c–5c, indicating moderate- to low-quality evidence mainly derived from observational studies and expert consensus. These findings support the principle of staged pulmonary rehabilitation for patients with CSCI, emphasizing that intervention timing should be tailored to dynamic changes in spinal cord edema, respiratory muscle function, and overall patient tolerance. Rehabilitation goals and training protocols across different phases need to be precisely matched to avoid secondary spinal cord injury caused by premature high-intensity training or missed opportunities for functional recovery due to delayed intervention (31, 32).

In the acute phase, interventions mainly target neural conduction disorders and respiratory muscle denervation caused by spinal cord edema (10, 24). Diaphragmatic breathing training and early electrical stimulation help maintain diaphragmatic contractility, activate residual motor units, and slow unfavorable muscle fiber type transformation. However, most supporting evidence remains low-level, lacking rigorous randomized controlled data. In the recovery phase, training strategies are designed to coincide with key windows of neuroplasticity (14). Progressive sitting training not only ameliorates orthostatic hypotension but also activates thoracolumbar sympathetic nerve function through postural changes, promoting synaptic reorganization between respiratory centers and spinal motor neurons (33). The 50% of maximal inspiratory pressure intensity adopted in resistive breathing training is based on the recruitment characteristics of diaphragmatic fibers—this intensity effectively activates type II muscle fiber metabolism while avoiding microdamage caused by excessive load (16). Chronic phase rehabilitation focuses on the systematic establishment of neural compensatory mechanisms (21). Glossopharyngeal insufflation training enhances coordination between cranial nerves and spinal cord-innervated muscle groups, progressively forming novel respiratory control patterns (15). The observed improvement in diaphragmatic coordination during virtual reality training may be associated with functional activation of the mirror neuron system and remodeling processes in the sensorimotor cortex (15). This combined mechanism of central regulation and peripheral effects provides important insights into the long-term recovery of respiratory function following CSCI. Notably, recovery mechanisms differ among patients with varying injury levels. Functional recovery in patients with C1-C2 injuries relies more on the activation of central pattern generators induced by diaphragmatic electrical stimulation, whereas patients with C3-C4 injuries may achieve functional improvement through compensatory enhancement of local cervical spinal cord neural networks. These differences suggest that rehabilitation protocols should be individualized based on the neuroanatomical characteristics of the injury level (6, 34).

Compared with conventional clinical practice, many institutions still adopt relatively uniform training regimens without clear staging or individualization according to injury level. Such undifferentiated implementation often fails to align with the temporal pattern of neural repair, thereby restricting the overall effect of functional recovery. Given the moderate-to-low quality of current evidence, the clinical application of staged rehabilitation should be performed cautiously with full consideration of patient safety and neurological status. Future studies are warranted to conduct high-quality RCTs to clarify optimal timing, intensity, and modality in each phase. Further research is also needed to explore the neurobiological mechanisms underlying stage-specific interventions and to establish standardized and individualized protocols based on injury level and neurological prognosis.

### 4.4 Position management varies across different stages of pulmonary rehabilitation

Evidence 12–13 in this review were graded as moderate- to low-level evidence, mostly based on observational research and physiological studies rather than large-scale RCTs. These pieces of evidence summarize the core role of position management in pulmonary rehabilitation for patients with CSCI. As an important adjuvant strategy, position management acts not only through gravity-related changes in respiratory mechanics but also by facilitating adaptive remodeling of the respiratory control system after spinal cord injury.

In the acute phase, supine positioning increases functional residual capacity by 15–20% by shifting abdominal contents cephalad to assist diaphragmatic movement, which is especially valuable for individuals at risk of respiratory muscle weakness (6). Alternating lateral decubitus positioning improves ventilation in dependent lung zones by approximately 30% and helps prevent atelectasis (35). However, the supporting evidence for these acute-phase positional strategies remains low- to moderate-level, lacking high-quality comparative clinical data. During the recovery phase, gradual elevation from supine to sitting posture enhances chest expansion and activates thoracic proprioceptive afferent pathways, supporting neuroplasticity. In the chronic phase, wheelchair positioning at a backrest angle of 100°–110° reduces accessory respiratory muscle activity and improves respiratory efficiency (16, 17). Customized bracing for patients with thoracic deformities further optimizes chest wall mechanics and proprioceptive feedback. Although these findings are physiologically meaningful, most derive from small-sample mechanistic studies, without robust high-level evidence confirming long-term clinical benefits.

Compared with routine clinical practice, many medical settings apply relatively fixed positional protocols without stage-based adjustment or individualization according to injury level. Such generalized application often fails to fully exploit the beneficial effects of posture on respiratory mechanics, hemodynamics, and neural remodeling. Due to the overall low-to-moderate quality of current evidence, clinical implementation should emphasize safety, spinal stability, and patient tolerance. Future research is needed to conduct high-quality RCTs to verify the efficacy and safety of staged position management. Further studies using functional imaging modalities such as fMRI are also warranted to clarify how different postures modulate brainstem respiratory center activity and respiratory neuroplasticity. Standardized, individualized positional protocols should be established based on neurological injury level to improve clinical applicability and therapeutic effects.

### 4.5 Diverse training content and variable frequency in pulmonary rehabilitation

Evidence 14–20 in this review summarizes pulmonary rehabilitation training for CSCI patients, with most evidence graded low to moderate quality, mainly from physiological studies, observational trials and expert consensus. Current findings support a diversified training program tailored to respiratory muscle function, injury level, rehabilitation stage and individual tolerance, with appropriate frequency and intensity to avoid overfatigue or inefficient monotonous training. A systematic regimen helps comprehensively improve respiratory muscle strength, endurance, coordination and airway clearance.

The physiological basis of respiratory muscle training lies in neural innervation remodeling and muscle fiber metabolic adaptation. Diaphragmatic breathing training enhances contraction amplitude, increasing tidal volume, an improvement attributed to both increased motor unit recruitment and altered abdominal pressure gradients that stretch lung parenchyma. Pursed-lip breathing, with an inspiratory-to-expiratory ratio of 1:2–3, prolongs expiration to maintain small airway opening pressure, thereby increasing functional residual capacity and preventing end-expiratory alveolar collapse (10, 11). The graded progression of resistance training follows skeletal muscle adaptation principles. Initial training at 50% of maximal inspiratory pressure targets fast- and slow-twitch fiber recruitment thresholds; increasing intensity to 70–80% has been associated with increase in diaphragmatic type II fiber cross-sectional area, reflecting enhanced muscle protein synthesis. The use of 0.5–1 kg loads in sandbag-assisted training optimizes the diaphragmatic length–tension relationship, improving contraction efficiency by approximately 30% (18). Regarding neurophysiological mechanisms, external diaphragmatic pacing activates residual phrenic nerve motor fibers to induce synchronized contractions. Four weeks of electrical stimulation has been shown to increase acetylcholine receptor density at motor endplates, indicating improved neuromuscular junction plasticity. Targeted neuromuscular electrical stimulation of intercostal nerves may activate intrinsic spinal neural networks, promoting coordinated respiratory movements above and below the injury level (26). Airway clearance techniques involve multiple physiological regulations. The active cycle of breathing technique cyclically alters intrathoracic pressure, increasing airway mucosal ciliary beat frequency (15). The CoughAssist device generates negative pressure peaks up to −40 cm H₂O, facilitating secretion mobilization and stimulating cough reflex arc integrity. For patients with C3–C5 injuries, device-assisted coughing increases peak cough flow to two to three times that of voluntary coughing, underscoring the compensatory role of assisted ventilation in respiratory muscle function.

Compared with conventional clinical practice, many institutions still adopt relatively simplified and uniform training protocols without individualized adjustment or staged progression, which often leads to suboptimal improvement in respiratory function and restricted long-term outcomes. Given the overall low-to-moderate quality of current evidence, clinical implementation should prioritize patient safety, neurological stability and stage-based training progression. Future research should carry out high-quality RCTs to determine the optimal training intensity, duration and combined regimens. Further neurophysiological and imaging studies are also warranted to clarify the mechanisms of training-induced neural and muscular remodeling, so as to establish more standardized and individualized pulmonary rehabilitation protocols for CSCI patients.

### 4.6 Systematic exercise training is essential for pulmonary rehabilitation beyond respiratory muscle training

Evidence 21–27 in this review was graded as low to moderate quality, mostly derived from physiological studies, observational research and expert consensus, with few high-quality RCTs. These findings indicate that effective pulmonary rehabilitation for CSCI patients requires comprehensive exercise training beyond isolated respiratory muscle work, targeting disorders of neuromuscular control and respiratory-motor coordination.

The mechanisms by which exercise training improves respiratory function operate at three levels: neural regulation remodeling, biomechanical optimization, and metabolic adaptation. At the neural level, upper limb resistance training activates residual corticospinal tracts to reinforce synergistic activation between accessory respiratory muscles and core muscle groups; for patients with C5–C8 injuries, 1–2 kg loads can increase electromyographic activity of the pectoralis major by approximately 40% while avoiding compensatory postural abnormalities (24). Aerobic training such as wheelchair propulsion not only improves cardiopulmonary function but also restores automatic respiratory rhythm regulation during movement, enhancing the respiratory center’s sensitivity to carbon dioxide and thus elevating exercise ventilatory efficiency (23). Traditional Chinese medicine techniques including the Six Healing Sounds and acupuncture offer holistic regulatory effects by modulating laryngeal muscle tension, vagal tone, and medullary neurotransmitter balance, providing multi-target support when integrated with modern rehabilitation. Virtual reality training improves respiratory-postural coordination by reinforcing the vestibulo-respiratory reflex and increasing functional connectivity between the motor cortex and brainstem respiratory centers by roughly 30% (24). Rib mobilization and spinal mobility training enhance costovertebral joint mobility, expand thoracic diameter by 10–15%, and optimize trunk mechanical alignment to reduce respiratory energy expenditure, with training frequency dynamically adjusted across acute, recovery, and chronic phases to match neuroplasticity-dependent synaptic remodeling. Measured improvements in thoracic expansion and exercise endurance ultimately reflect optimized intercostal muscle mechanics and enhanced mitochondrial oxidative phosphorylation efficiency (19, 22).

Compared with conventional clinical practice, many rehabilitation programs focus only on local respiratory muscle training and neglect whole-body exercise coordination and central neural remodeling. Such single-modal training often results in incomplete functional recovery and limited improvement in exercise capacity. Given the overall low-to-moderate quality of current evidence, clinical application should prioritize safety, neurological stability and individualized progression. Future research should conduct high-quality RCTs to verify the efficacy of comprehensive exercise training and determine optimal intensity, frequency and duration. Multimodal neuroimaging studies are warranted to clarify neural plasticity mechanisms within spinal circuits above and below the injury level, supporting the development of standardized, evidence-based rehabilitation protocols for respiratory-motor coordination in CSCI patients.

### 4.7 Effective pulmonary rehabilitation health education strategies can improve patients’ self-management abilities and reduce complications

Evidence 28–29 in this review were graded as low- to moderate-quality evidence, mainly from observational and small-sample clinical studies, with limited high-level randomized controlled data. These findings indicate that health education serves as a key component in pulmonary rehabilitation for CSCI patients, acting not only through knowledge transmission but also by modulating neuropsychological processes and facilitating adaptive neural changes after injury (21).

In terms of skill training, instructional methods combining visual demonstrations with tactile feedback strengthen functional connections between the parietal sensory cortex and the premotor area. This multisensory learning approach enhances the acquisition of motor skills, which relates to the coordinated processing of sensorimotor information. The “simulated operation assessment” training model for family caregivers is designed based on the positive influence of the action observation neural network on practical operational abilities (23). Individualized education programs must account for differences in neuroplasticity among patients. Variations in brain functional connectivity patterns exist among patients with different educational backgrounds, necessitating the adaptation of educational materials to diverse information-processing styles. For example, individuals with strong visuospatial abilities benefit more from illustrated materials, whereas those with stronger language skills may be better suited to textual instructions (25). The significance of long-term follow-up mechanisms extends beyond information updates. Regular assessments and feedback constitute a dynamic reinforcement system that helps sustain patients’ self-management behaviors through dopaminergic reward pathways. The establishment of rehabilitation records not only documents disease progression but also promotes the conversion of explicit memory to procedural memory, facilitating the internalization of rehabilitation skills (6, 14). The development of family support systems reflects fundamental principles of social neuroscience. Activities within family support groups activate the brain’s default mode network, which is associated with improved psychological adaptability. Effective management of caregivers’ psychological stress not only enhances care quality but also indirectly regulates patients’ autonomic nervous function balance through emotional transmission mechanisms (36). The transition from passive reception to active participation in health education essentially reflects adjustments in the brain’s executive function network. When patients participate in setting rehabilitation goals, functional connectivity between the dorsolateral prefrontal cortex and basal ganglia strengthens, indicating improved cognitive control. Self-monitoring training helps enhance error detection functions of the anterior cingulate cortex, thereby improving patients’ awareness of changes in their physiological signals (37).

Compared with conventional clinical practice, many institutions only provide fragmented, one-way knowledge instruction without individualized design, staged promotion, or family-centered training. Such passive education leads to poor adherence and limited improvement in self-management. Given the overall low-to-moderate quality of supporting evidence, clinical application should emphasize systematic, interactive, and personalized health education strategies. Future research should conduct high-quality RCTs to evaluate the long-term efficacy of standardized health education programs. Further studies using neuroimaging and functional connectivity analysis are also warranted to explore how education modulates brain network plasticity, thereby supporting the development of more targeted, neuroscience-informed pulmonary rehabilitation education strategies.

### 4.8 Clinical recommendations

Based on the best evidence summarized in this review and the corresponding evidence levels, the following clinical recommendations are provided for adult patients with CSCI (Table 5). Interventions supported by high-quality evidence (Level 1a–1b), including individualized pulmonary function assessment and threshold inspiratory muscle training at 50% of maximal inspiratory pressure, are strongly recommended as core components. Interventions supported by moderate-quality evidence (Level 2c–3), such as staged rehabilitation and staged position management, are recommended with ongoing monitoring. Adjunctive options supported by lower-quality evidence (Level 4–5b), including multidisciplinary team involvement, singing training, TCM techniques, and family-centered health education, may be offered based on patient preference and available resources. All protocols should be individualized by injury level, ASIA grade, and baseline function, with reassessment every 6 weeks.

**Table 5:** Clinical recommendations for pulmonary rehabilitation in adult patients with CSCI.

Recommendation category	Evidence level	Recommendations (simplified)
Strongly recommended	Level 1a–1b	1. Individualized baseline assessment (FVC, FEV1, PEFR, DLCO, respiratory impedance) 2. Threshold inspiratory muscle training at 50% MIP
Recommended	Level 2c–3	1. Staged rehabilitation: bedside training in acute phase, out-of-bed in recovery phase 2. Staged position management: supine → sitting transition
Adjunctive options	Level 4–5b	1. Multidisciplinary team collaboration 2. Singing training / TCM pulmonary rehab / Music therapy 3. Family-centered health education
General principles	Expert consensus	1. Individualize protocols by injury level, ASIA grade, time since injury, baseline function 2. Reassess every 6 weeks to adjust training

CSCI, Cervical spinal cord injury; FVC, Forced vital capacity; FEV1, Forced expiratory volume in one second; PEFR, Peak expiratory flow rate; DLCO, Diffusion capacity of the lungs for carbon monoxide; MIP, Maximal inspiratory pressure; TCM, Traditional Chinese medicine; ASIA, American Spinal Injury Association.

### 4.9 Limitations

Several limitations of this review should be acknowledged. First, the quality of the included evidence is variable, with the majority of recommendations derived from low- or moderate-quality studies (Level 2c–5b). This reflects inherent challenges in CSCI research, including small sample sizes, heterogeneity of injury levels, and difficulties in blinding rehabilitation interventions. Second, this review focused exclusively on adult patients (aged ≥18 years); findings may not be generalizable to pediatric populations. Additionally, most included studies reported short-term outcomes (≤6 weeks), with limited data on long-term sustainability of rehabilitation gains. Third, most included studies were from Chinese databases, introducing potential geographic bias. In Chinese clinical practice, TCM-based interventions are considered first-line or adjunctive treatments for CSCI, an advantageous approach for this condition. Therefore, our findings are primarily applicable to Chinese healthcare settings. Generalizability to other regions or systems requires further validation through international multicenter studies.

## 5 Conclusion

Pulmonary rehabilitation for patients with CSCI requires multidisciplinary collaboration, phased strategies, dynamic assessment, and individualized protocols. Rehabilitation approaches should be tailored to resource availability, team expertise, and patient characteristics rather than applying standardized protocols. Several challenges remain. Technologically, portable assessment devices and digital tools are underutilized, especially in primary care. Professionally, shortages of respiratory therapists persist, and some providers narrowly focus on respiratory muscle training alone. Regarding standards, existing guidelines lack localized research data from Chinese populations. Future efforts should focus on three areas: developing cost-effective rehabilitation equipment and internet-based models; enhancing personnel training through systematic education; and conducting multicenter studies to establish Chinese-specific rehabilitation standards. Additionally, community-hospital-enterprise collaboration should support patients’ transition from functional improvement to social participation.

## Data Availability

The original contributions presented in the study are included in the article/Supplementary material, further inquiries can be directed to the corresponding author.
