# Supplementary material for: Best-evidence summary of pulmonary rehabilitation strategies for adult patients with cervical spinal cord injury: a systematic review
Source: Front Med (Lausanne). 2026 Sep 22;13:1817133. doi: 10.3389/fmed.2026.1817133 (PMC13638520; doi:10.3389/fmed.2026.1817133)
Supplement: Supplementary file 1 [file Table_1.DOCX]

Table S1. Search strategy for Web of Science

| Steps | Retrieval formula |
| --- | --- |
| #1 | TS=(("spinal cord injury" OR "spinal cord injuries" OR paraplegia OR "spinal fracture" OR "spinal trauma" OR "cervical spinal cord injury" OR tetraplegia OR quadriplegia)) |
| #2 | TS=(("respiratory tract" OR "respiratory management" OR "pulmonary rehabilitation" OR "respiratory function training" OR "respiratory training" OR "respiratory support" OR "breathing exercise" OR "respiratory muscle training")) |
| #3 | #1 AND #2 |
| #4 | #3 AND DT=(Article OR Review) AND LA=(English) |

Table S2. Search strategy for Embase

| Steps | Retrieval formula |
| --- | --- |
| #1 | 'spinal cord injury'/exp OR 'paraplegia'/exp OR 'quadriplegia'/exp |
| #2 | 'cervical spinal cord injury':ab,ti OR 'spinal fracture':ab,ti OR 'spinal trauma':ab,ti |
| #3 | #1 OR #2 |
| #4 | 'lung rehabilitation'/exp OR 'respiratory muscle training'/exp |
| #5 | 'pulmonary rehabilitation':ab,ti OR 'respiratory training':ab,ti OR 'breathing exercise':ab,ti OR 'respiratory support':ab,ti |
| #6 | #4 OR #5 |
| #7 | #3 AND #6 |
| #8 | #7 AND ([article]/lim OR [review]/lim) AND [english]/lim |

Table S3. Search strategy for the Cochrane Library

| Steps | Retrieval formula |
| --- | --- |
| #1 | MeSH descriptor: [Spinal Cord Injuries] explode all trees |
| #2 | (paraplegia OR spinal fracture OR spinal trauma OR cervical spinal cord injury OR tetraplegia):ti,ab,kw |
| #3 | #1 OR #2 |
| #4 | MeSH descriptor: [Respiratory Therapy] explode all trees |
| #5 | (pulmonary rehabilitation OR respiratory training OR breathing exercise OR respiratory muscle training):ti,ab,kw |
| #6 | #4 OR #5 |
| #7 | #3 AND #6 |

Table S4. Search strategy for CNKI (English translation)

| Steps | Retrieval formula |
| --- | --- |
| #1 | SU=('spinal cord injury' OR 'cervical spinal cord injury' OR 'paraplegia' OR 'quadriplegia' OR 'spinal fracture' OR 'spinal trauma') |
| #2 | SU=('pulmonary rehabilitation' OR 'respiratory training' OR 'respiratory management' OR 'breathing training' OR 'respiratory support' OR 'lung rehabilitation') |
| #3 | #1 AND #2 |
| Limits | Document type: Journal articles, dissertations; Language: Chinese |

Table S5. Search strategy for Wanfang Data (English translation)

| Steps | Retrieval formula |
| --- | --- |
| #1 | Title or Keyword: ("spinal cord injury" OR "cervical spinal cord injury" OR "paraplegia" OR "quadriplegia" OR "spinal fracture") |
| #2 | Title or Keyword: ("pulmonary rehabilitation" OR "respiratory training" OR "respiratory management" OR "breathing exercise" OR "respiratory support") |
| #3 | #1 AND #2 |
| Limits | Document type: Journal articles, dissertations; Language: Chinese |

Table S6. Search strategy for SinoMed (English translation)

| Steps | Retrieval formula |
| --- | --- |
| #1 | (spinal cord injury OR cervical spinal cord injury OR paraplegia OR quadriplegia OR spinal fracture OR spinal trauma) [Common Field] |
| #2 | (pulmonary rehabilitation OR respiratory training OR respiratory management OR respiratory support OR breathing exercise) [Common Field] |
| #3 | #1 AND #2 |
| Limits | Document type: Journal articles; Language: Chinese |

Table S7. Search strategy for CNKI (Chinese version)

| Steps | Retrieval formula |
| --- | --- |
| #1 | SU=('脊髓损伤' OR '颈椎损伤' OR '截瘫' OR '四肢瘫痪' OR '脊柱骨折' OR '脊柱创伤') |
| #2 | SU=('肺康复' OR '呼吸训练' OR '呼吸管理' OR '呼吸锻炼' OR '呼吸支持') |
| #3 | #1 AND #2 |
| 限定 | 文献类型：期刊论文、学位论文；语种：中文 |

Table S8. Search strategy for Wanfang Data (Chinese version)

| Steps | Retrieval formula |
| --- | --- |
| #1 | (脊髓损伤 OR 颈椎损伤 OR 截瘫 OR 四肢瘫痪 OR 脊柱骨折 OR 脊柱创伤) [常用字段] |
| #2 | (肺康复 OR 呼吸训练 OR 呼吸管理 OR 呼吸支持 OR 呼吸锻炼) [常用字段] |
| #3 | #1 AND #2 |
| 限定 | 文献类型：期刊论文；语种：中文 |

Table S9. Search strategy for SinoMed (original Chinese version)

| Steps | Retrieval formula |
| --- | --- |
| #1 | (脊髓损伤 OR 颈椎损伤 OR 截瘫 OR 四肢瘫痪 OR 脊柱骨折 OR 脊柱创伤) [常用字段] |
| #2 | (肺康复 OR 呼吸训练 OR 呼吸管理 OR 呼吸支持 OR 呼吸锻炼) [常用字段] |
| #3 | #1 AND #2 |
| 限定 | 文献类型：期刊论文；语种：中文 |
